# Comparing the Effects of Lime Soil and Yellow Soil on Cadmium Accumulation in Rice during Grain-Filling and Maturation Periods

**DOI:** 10.3390/plants13152018

**Published:** 2024-07-23

**Authors:** Hu Wang, Lang Teng, Xu Mao, Tengbing He, Tianling Fu

**Affiliations:** 1 Institute of New Rural Development, Guizhou University, Guiyang 550025, China; wanghu011@126.com (H.W.); hetengbing@163.com (T.H.); 2 Guizhou Chuyang Ecological Environmental Protection Technology Co., Ltd., Guiyang 550025, China

**Keywords:** Cd, accumulation, rice, karst, ion, soil type

## Abstract

The karst area has become a high-risk area for Cadmium (Cd) exposure. Interestingly, the high levels of Cd in soils do not result in an excessive bioaccumulation of Cd in rice. Carbonate rock dissolution ions (CRIs) could limit the accumulation and translocation of Cd in rice. CRIs can become a major bottleneck in the remediation and management of farmlands in karst areas. However, there is limited research on the effects of CRIs in soils on Cd accumulation in rice. The karst area of lime soil (LS) and the non-karst areas of yellow soil (YS) were collected, and an external Cd was added to conduct rice cultivation experiments. Cd and CRIs (Ca^2+^, Mg^2+^, CO_3_^2−^/HCO_3_^−^, and OH^−^) in the rice–soil system were investigated from the grain-filling to maturity periods. The results showed that CRIs of LS were significantly higher than that of YS in different treatments. CRIs of LS were 2.05 mg·kg^−1^ for Ca^2+^, 0.90 mg·kg^−1^ for Mg^2+^, and 42.29 mg·kg^−1^ for CO_3_^2−^ in LS. CRIs could influence DTPA Cd, resulting in DTPA Cd of LS being lower than that of YS. DTPA Cd of YS was one to three times larger than that of YS. Cd content in different parts of rice in YS was higher than that of LS. Cd in rice grains of YS was one to six times larger than that of LS. The uptake of Cd from the soil during Filling III was critical in determining rice Cd accumulation. CRIs in the soil could affect Cd accumulation in rice. Ca^2+^ and Mg^2+^ had significant negative effects on Cd accumulation of rice at maturity and filling, respectively. CO_3_^2−^/HCO_3_^−^ and OH^−^ had significant negative effects on DTPA Cd in soil.

## 1. Introduction

Soil pollution by heavy metals has been both serious and widespread, which has become an unstable factor that affects crop production and food safety [1]. For example, the mean concentration of Cd in rice of Hunan (0.49 mg/kg), Guangdong (0.42 mg/kg), Guizhou (0.33 mg/kg), and Jiangxi (0.3 mg/kg) were higher than the food safety screening values (0.2 mg/kg) (GB 2762–2022) in China [2]. Among various heavy metals, Cd is of particular concern because of its high mobility, high toxicity, easy accumulation, and difficulty in elimination [3,4]. The National Soil Pollution Survey Bulletin issued by the Chinese government in 2014 indicated that the farmland soil pollutants at 19.4% of points exceeded the standard, Cd in 7.0% of the points exceeded the standard rate [5], and Cd was concentrated in the southwestern karst region (e.g., Guizhou, Guangxi, Chongqing). In China, it is also estimated that approximately 10 million hm^−2^ of contaminated farmland and 3.33 million hm^−2^ of farmlands are unsuited for farming due to pollution [2]. The enrichment of Cd in the soil is usually also derived from the parent rock [6]. Previous studies have shown that the soil Cd in carbonate, clasolite, and quaternary sediment regions exceeded the risk screening values of 70.77%, 6.89%, and 18.75%, respectively, in Guangxi, among which the carbonate rock region exceeded the limit most seriously [7]. However, the rice Cd exceeded the national standard in carbonate, clasolite, and quaternary sediment regions were 2%, 13.79%, and 9.38%, respectively [7]. Soil derived from carbonate rocks has higher total Cd and lower mobile Cd proportion than soil from the non-karst areas (clasolite and quaternary sediment regions), which had little impact on the quality risk of crops [7,8,9]. Similarly, the high levels of Cd in potato-/rice-planting soils would not result in an excessive bioaccumulation of Cd in Guizhou [9,10]. Therefore, Cd concentration in crops was lower in karst areas with a high Cd background (0.65 mg·kg^−1^).

There is a prominent issue of soil Cd exceeding the standard in the karst region in China. The standards of soil Cd were 0.3 (pH ≤ 5.5), 0.4 (5.5 < pH ≤ 6.5), 0.6 (6.5 < pH ≤ 7.5), and 0.6 (pH > 7.5) mg·kg^−1^ based on soil environmental quality risk control standards for soil contamination of agricultural land (GB 15618–2018) in China [9]. However, the soil Cd activity in this region is low, and rice exceeding the national standard is much lower than that in non-karst areas [6,7,9,11,12,13]. Pu et al. [14] and Fang et al. [15] found that most soil samples in the karst region were rich in CRIs. It was demonstrated that Cd^2+^ in the soil can form CdCO_3_ precipitation, Cd(OH)_2_ precipitation, and (Cd, Ca)CO_3_ coprecipitation with Ca^2+^, CO_3_^2−^, and OH^−^ under alkaline conditions, reducing the activity of Cd in the soil [16,17,18]. Moreover, Ca^2+^, Mg^2+^, and Cd^2+^ have the same positive charge and specific surface area and can compete with Cd for plant uptake. Ca and Mg are among the 17 essential nutrients for plant growth [19]. They are active in plants and inhibit Cd migration in plants [20,21]. The supplementation of both Ca and Mg decreased Cd accumulation and translocation in rice tissues in Cd-polluted soil [8]. It is inferred that the typical carbonate erosion in the karst region forms the rich components of CRIs, which plays an important role in regulating the migration, absorption, and trans-shipment of Cd in the soil–water–crop system.

The karst area has become a high-risk area for Cd exposure [9]. In recent years, several methods have been studied to control Cd risks in karst areas, such as water management, fertilization, physical treatments, chemical remediation via the addition of soil amendments, bioremediation, and phytoremediation [8]. Moreover, a large number of pilot demonstration projects to treat and restore contaminated soil were launched. However, soil derived from carbonate rocks has higher total Cd and lower mobile Cd proportion [7]. Li et al. [7] found that soil carbonates raised soil pH of Cd, significantly reducing the bioavailability of Cd in karst areas. The classification of agricultural land environmental quality and agricultural land safe usage based on Cd content in the soil has a wide deviation. CRIs can become a major bottleneck in the remediation and management of farmlands in karst areas, thus resulting in immeasurable ecological risk and waste of cultivated land resources.

In this study, the karst area of LS and the non-karst areas of YS were collected, and an external Cd was added to conduct rice cultivation experiments. The primary aims of this work were to (1) analyze the effects of dynamic change in CRI content in the root-zone soil on Cd accumulation in rice during grain-filling and maturation Periods; (2) explore the inhibitory effects of CRIs in the soil on Cd accumulation during the rice filling–ripening period. However, there is limited research on the effects of CRIs in soils on Cd accumulation in rice. The results of this study will provide a theoretical basis for the safe utilization of Cd-contaminated soils in karst areas and safe rice production.

## 2. Materials and Methods

### 2.1. Plant Materials and Experimental Design

The experiment used soil samples collected from Huaxi District, Guizhou Province. LS was derived from dolomite rocks. YS was derived from mudstone rocks. LS and YS were collected from a surface (0–20 cm) of farmland soil. The basic physical and chemical properties of YS and LS are shown in Table 1. The collected soil was transported to a laboratory for natural air drying, and large gravel, stone, and branches were removed. The soil was ground through a 2 mm sieve and mixed thoroughly for later use.

In 2022, a rice pot experiment was conducted at the Guiyang Comprehensive Test Station of the Guizhou Academy of Agricultural Sciences, Guizhou, China. Each pot (15 cm × 20 cm) was filled with 20 kg air-dried YS and LS, respectively. An external CdCl_2_ was added in LS and YS to conduct rice cultivation experiments. CdCl_2_ treatments in YS/LS were designed as 0.72, 1.65, 3.25, and 4.20 mg·kg^−1^, regarded as YS1/LS1, YS2/LS2, YS3/LS3, and YS4/LS4, respectively. Each treatment was repeated three replicates. The pot experiment was completely randomized with three replicates and a total of 24 pots. The soil had a balance period of 60 d. Base fertilizers were applied and thoroughly mixed with soil, filled with water until they reached saturation, and allowed to equilibrate for 7 d. The base fertilizer used was (NH_4_)_3_PO_4_, with a phosphorus pentoxide content of 210 mg·kg^−1^, urea with a nitrogen content of 280 mg·kg^−1^, and K_2_CO_3_ with a K_2_O content of 200 mg·kg^−1^. Rice was planted. The rice cultivar, C Liangyouhuazhan, was obtained from the Rice Research Institute, Guizhou Academy of Agricultural Sciences. During the tillering stage, 5 g/pot (equivalent to 41.7 kg·hm^−2^) of urea (N ≥ 46.2%) was applied as topdressing. During the early filling stage, 5 g/pot (equivalent to 41.7 kg·hm^−2^) of compound fertilizer (N:P:K = 16:8:18) was applied as topdressing.

### 2.2. Sample Collection and Preparation

Rice grain filling is the most important physiological process of grain formation, and it is also the decisive stage that determines grain weight, yield, and rice quality [22]. In this study, rice grain filling was measured at the first week of filling (Filling I), the second week of filling (Filling II), and the third week of filling (Filling III). Soil and rice plant samples were collected as follows: Filling I (6 September), Filling II (13 September), Filling III (20 September), and maturity (27 September, the maturity stage). There were the 96-soil sample and the 312-rice plant sample. Soil samples were air-dried in the laboratory and separated by using the coning and quartering method. They were then passed through a 2 mm and 0.149 mm nylon sieve for subsequent analyses. Rice plant samples were collected and transported to the laboratory. The plants were washed with tap water to remove the soil on the roots and then rinsed three times with ultrapure water. They were then separated into five parts: roots, stems, leaves, cobs, and grains. The separated parts were oven-dried at 105 °C for 2 h and then at 70 °C until reaching a constant weight. The grains were dehulled to obtain the glumes and brown rice samples, which were ground into powder using an agate mortar and sieved through a 0.425 mm nylon sieve for subsequent analyses.

### 2.3. Chemical Analysis and Quality Control

Soil pH was determined using a distilled water extraction (soil/water ratio of 1:2.5) by the potentiometric method. Soil total alkalinity (CO_3_^2−^/HCO_3_^−^) was determined by the acid–base titration method with a 1:5 soil/water ratio. A total of 0.1 g of soil sample was added to a high-pressure airtight tank and digested for total Cd, Ca, and Mg with HF (1 mL)–HNO_3_ (3 mL)–HClO_4_ (1 mL) by the autoclave at a constant temperature in the drying oven [9]. The bioavailability of Cd (DTPA-Cd) was extracted by DTPA (extraction in 5 mM diethylenetriaminepentaacetic acid). Ca^2+^ and Mg^2+^ were extracted by ultrapure water at a solid–liquid ratio of 1:5.

The sample trace elements were determined as follows: 0.2 g of the sample was added to 5 mL of nitric acid and digested in a graphite digestion instrument at 120 °C for 2 h. The digestion was complete when no white precipitate was observed in the digestion tank. The temperature was then adjusted to 150 °C to evaporate the acid to the size of soybean grains. High-purity reagents and ultrapure water were used throughout the experiment. The analysis of soil sample and rice plant sample had two replicates.

Cd content was determined by inductively coupled plasma-optical emission spectroscopy (ICP-OES-7400, Thermo Fisher Scientific, Waltham, MA, USA). Soil Ca^2+^ and Mg^2+^ were determined by atomic absorption spectrophotometry (ICE-3500, Thermo Fisher Scientific, USA).

The quality of Cd in the soil samples was controlled using the standard soil sample (Number: GBW07405) from the National Center for Standard Materials, China. The standard soil sample was as follows: Cd 0.39–0.51 mg·kg^−1^. The quality of Cd, Ca, and Mg in the rice plant samples was controlled using the plant standard sample (Number: GBW10048) from the National Center for Standard Materials, China. The standard plant samples were as follows: Cd 0.086–0.098 mg·kg^−1^, Ca 1.60–1.72 g·kg^−1^, and Mg 0.50–0.56 g·kg^−1^. Blank reagent tests were performed, and the sample recovery rate was between 90% and 110%.

### 2.4. Measurement of Photosynthetic Parameters

The photosynthetic parameters of rice were measured by using a portable chlorophyll meter (SPAD-502 Plus, Minolta, Tokyo, Japan) [23]. An intact leaf from three rice plants with uniform growth conditions for each plant was selected during grain-filling and maturation periods, the SPAD value was measured at the central position six times, and the average value was taken as the SPAD value for that point.

### 2.5. Statistical Analyses

Data sorting was performed using Excel 2019. Normal distribution test, one-way ANOVA, and Pearson correlation analysis were conducted using SPSS 22.0. Origin 2018 software was used for plotting.

## 3. Results

### 3.1. DTPA Cd Content in the Soil during Rice Filling and Maturity

The DTPA Cd of YS was higher than that of LS in different treatments (Table 2), indicating that soil derived from carbonate rocks had a lower mobile Cd proportion. DTPA Cd of YS was one to three times larger than that of YS. The DTPA Cd increased with the increase in Cd treatments. The DTPA Cd of LS and YS in different treatments increased from Filling I to Filling III and decreased from Filling III to maturity and had the highest activity in Filling III. The DTPA Cd contents for LS1, LS2, LS3, and LS4 in Filling III were 0.122, 0.473, 0.686, and 1.432 mg·kg^−1^, respectively. The DTPA Cd contents for YS1, YS2, YS3, and YS4 in Filling III were 0.288, 0.654, 1.013, and 2.053 mg·kg^−1^, respectively.

### 3.2. Ca and Mg Content in the Soil during Rice Filling and Maturity

**Figure 1:**
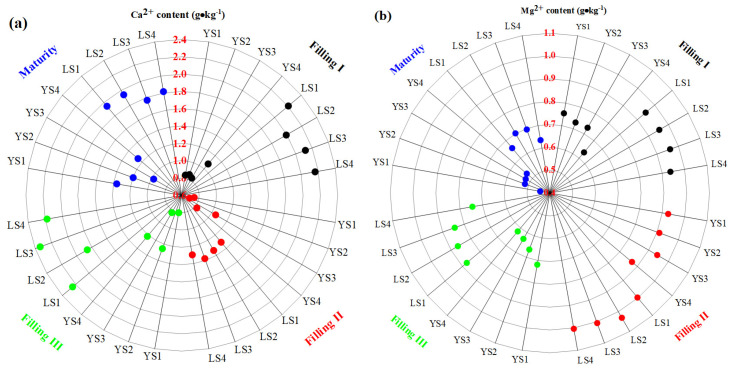
The Ca^2+^ (**a**) and Mg^2+^ (**b**) content of LS and YS in different treatments during rice filling and maturity.

The total Ca and Mg content of LS and YS in different treatments gradually decreased from rice filling to maturity, and the total Ca and Mg content in LS was significantly higher than YS (Table 3). The total Ca and Mg content of LS in different treatments had no significant difference, while YS had significant difference. Compared to Filling I, the decrease degrees of total Ca content for YS1, YS2, YS3, and YS4 in Filling III were 12.56%,11.62%, 34.46%, and 28.44%, respectively. Compared to Filling I, the decrease degrees of total Mg content for YS1, YS2, YS2, and YS2 in maturity were 30.92%, 30.78%, 42.45%, and 48.28%, respectively. The total Mg content of LS was roughly 1.7 times that of YS under the same treatment. There was an abundant karst component in LS, and formed CRIs (e.g., Ca, Mg), resulting in Ca^2+^ and Mg^2+^ in LS being higher than YS (Figure 1). Compared to Filling I, the increased degrees of Ca^2+^ for YS1, YS2, YS3, and YS4 in Filling III were 62.73%, 39.96%, 17.43%, and 17.46%, respectively. The Mg^2+^ content of LS and YS in different treatments could reach the highest level in Filling II and then decrease from Filling II to maturity. The increased degree of Mg^2+^ for YS from Filling I to mature became larger with the increase in Cd stress concentration. Mg^2+^ content in LS increased from Filling I to Filling II and decreased from Filling III to maturity.

### 3.3. Total Alkalinity Content and pH in the Soil during Rice Filling and Maturity

**Figure 2:**
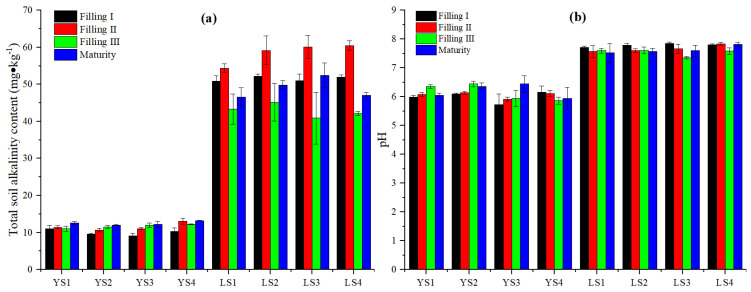
Total alkalinity content (**a**) and pH (**b**) of LS and YS in different treatments during rice filling and maturity.

The total alkalinity and pH of LS were significantly higher than that of YS in different treatments (Figure 2). Compared to Filling I, the total alkalinity of YS1, YS2, YS3, and YS4 was highest in maturity, which increased by 13.36%, 24.34%, 33.28%, and 28.95%, respectively. The total alkalinity of LS1, LS2, LS3, and LS4 reached the maximum in Filling II and the minimum in Filling III and decreased by 20.33%, 23.65%, 31.92%, and 30.31%, respectively, from Filling II to Filling III. The pH of soil increases with the increase in OH^−^ concentration. In LS1 and LS2 for the low Cd treatment, the pH could reach the highest level in Filling III and then decrease. In LS3 and LS4 for the high Cd treatment, the pH of YS3 increased from rice filling to maturity, and the pH of LS4 decreased. The pH of LS in different treatments was relatively stable during rice filling and maturity, except for a slight decrease in LS3 during filling, indicating that Cd treatments in LS reduced the OH^−^ concentration in Filling III.

### 3.4. Cd Content in Different Parts of Rice during Rice Filling and Maturity

**Figure 3:**
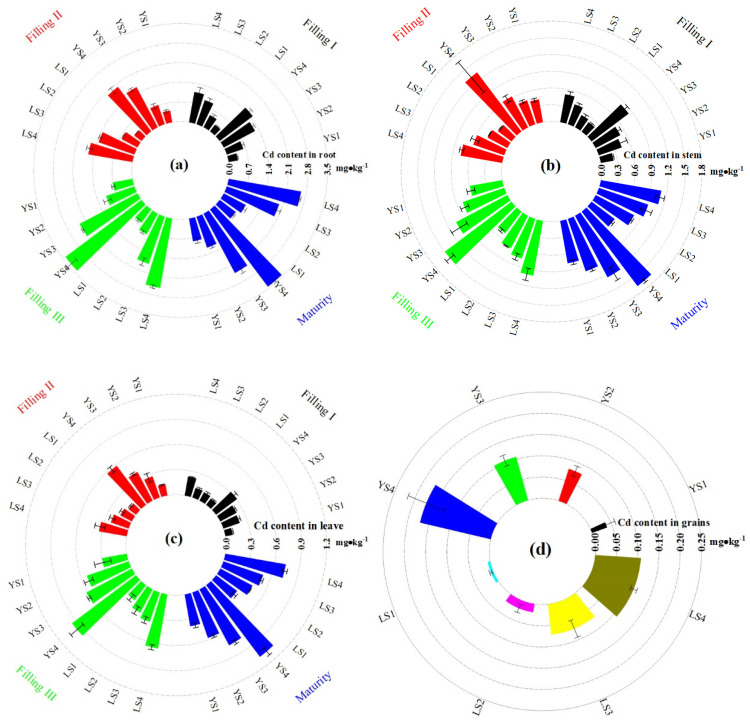
Cd content in root (**a**), stem (**b**), leaves (**c**), and grain (**d**) during rice filling and maturity.

The Cd content in different parts of rice in YS was significantly higher than that of LS (Figure 3). Cd content of different treatments was in the following order: roots > stems > leaves > grains. Cd content in different parts of rice increased with the increase in Cd treatments. The increased degree of Cd content in different parts of rice was the most obvious from Filling II to Filling III, indicating that Filling III is the key period of Cd accumulation in rice. The increased degree of Cd content in the roots slowed down from Filling III to maturity. The high levels of Cd in soils would not result in an excessive bioaccumulation of Cd in rice. The Cd content of LS was higher than that of YS in different parts of rice. Cd in rice grains of YS was one to six times larger than that of LS. Cd in rice grains of LS and YS in different treatments did not exceed the national standard (GB 2762-2017) [5]. However, Cd treatments could increase Cd in rice grains with the increase in Cd treatments, and the Cd content of LS4 and YS4 were 0.171 and 0.108 mg·kg^−1^, respectively.

### 3.5. Total Ca and Mg Content of Rice Plants in Different Treatments at Maturity

Total Ca and Mg content of rice plants in different treatments at maturity is shown in Table 4. Total Mg content in different parts of rice increased with the increase in Cd treatments. The total Mg content of rice plants in LS was significantly higher than YS. Total Ca content in root, stem, and leaves decreased with the increase in Cd treatments. Total Ca content in grain increased with the increase in Cd treatments. The total Ca content of root and leaves in LS was significantly higher than YS.

### 3.6. Correlation between CRI and DTPA Cd in Soil and Cd Content in Different Parts of Rice

**Figure 4:**
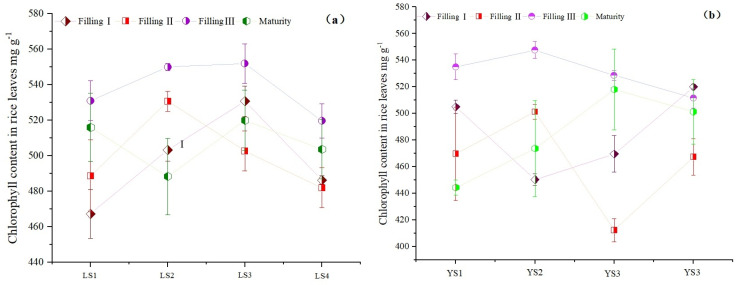
The chlorophyll content in leaves from LS (**a**) and YS (**b**) during rice filling and maturity.

As shown in Table 5, there was a significant negative correlation between CRI content and DTPA Cd, indicating that CRIs in soil could reduce the activity of Cd. There was a negative correlation between Ca^2+^ and Mg^2+^content in soil and Cd content in different parts of rice (Table 6). The regulatory role of Mg^2+^ on chlorophyll synthesis and degradation is also shown [24]. Photosynthesis of leaves was the highest at Filling III (Figure 4), indicating that Mg^2+^ played an important role in rice plants. The Cd content in rice stem leaves and grains had significantly negative effects on Mg^2+^ content in soil at Filling III, indicating that Mg^2+^ played an important role in inhibiting Cd in rice at Filling III, respectively. 

## 4. Discussion

Soil derived from carbonate rocks (e.g., dolomite, limestone) is rich in CRIs (e.g., Ca^2+^, Mg^2+^, OH^−^, and HCO_3_^−^) in the karst region [14,25,26]. The CRIs of YS were 0.55 mg·kg^−1^ for Ca^2+^, 0.43 mg·kg^−1^ for Mg^2+^, and 10.76 mg·kg^−1^ for CO_3_^2−^ in Guizhou (Table 1). CRIs of LS were significantly higher than that of YS in different treatments in this study (Table 1). However, our carbonate rock dissolution experiment is the first time to demonstrate that the varying soil CRIs, more importantly CRIs in LS during grain-filling and maturation periods, can reduce Cd accumulation in rice (Figure 3).

DTPA Cd of the karst area was higher than that of the non-karst areas in different treatments (Table 2). These results were the same as those observed by Li and Wei and indicated that soil derived from carbonate rocks had lower mobile Cd proportion [27,28]. However, many studies had only focused on pH playing an important role in decreasing DTPA Cd [29], ignoring the effect of CRIs. In this study, there was a negative correlation between CRI content and DTPA Cd (Table 5). Wang’s research showed that CO_3_^2−^/HCO_3_^−^, and OH^−^ can decrease DTPA Cd in soil [30]. Cd^2+^ can form CdCO_3_ precipitation, Cd(OH)_2_ precipitation, and (Cd, Ca)CO_3_ coprecipitation with Ca^2+^, CO_3_^2−^, and OH^−^ under alkaline conditions [17,31]. Meanwhile, CO_3_^2−^ can hydrolyze to generate OH^−^, which could form difficult-to-soluble Cd(OH)_2_ precipitate with Cd, thereby reducing the activity of Cd [32]. Moreover, the increase in soil pH reduces the solubility of heavy metals in the soil, increases the pH-dependent charge, and increases the adsorption of metals by soil particles [20]. HCO_3_^−^ in the soil mainly affects the activity of Cd by ionization to generate CO_3_^2−^ and hydrolysis to generate OH^−^. Ca^2+^ and Mg^2+^ compete with Cd for adsorption in the soil as a plant nutrient with the same volume and valence as Cd^2+^ [21,33]. The classification of agricultural land environmental quality and agricultural land safe usage based on Cd content in the soil has a wide deviation, especially in karst areas [9]. CRIs played an important role in decreasing DTPA Cd in karst areas. 

The Cd uptake from the soil during rice grain filling is critical for grain Cd concentrations [34]. It was found that the DTPA Cd of LS and YS in different treatments increased from Filling I to Filling III and then decreased from Filling II to maturity in this study. DTPA Cd of LS and YS in different treatments had the highest activity in Filling III. This might be related to the decrease in CO_3_^2−^/HCO_3_^−^. The total alkalinity (CO_3_^2−^/HCO_3_^−^) of LS1, LS2, LS3, and LS4 reached the minimum in Filling III in this study (Figure 2a). Ca^2+^ and Mg^2+^ were a component of plant cell walls and beneficial to the growth and development of plants [35,36]. As plant nutrient elements, Ca and Mg could inhibit the absorption of Cd by plants and reduce Cd toxicity in plants [8,37]. Ca^2+^ and Mg^2+^ were absorbed to inhibit the absorption of Cd, enhance the detoxification mechanism of rice, and improve its tolerance to Cd [8]. In this study, Mg^2+^ content in different parts of rice increased with the increase in Cd treatments (Table 4). There was a negative correlation between Ca^2+^ content in soil and Cd content in different parts of rice (Table 6). Ca^2+^ is preferentially absorbed by plants and restrained the Cd root-to-stem transport [38]. There was increased Ca^2+^ in different parts of rice, effectively preventing Cd transportation from roots to the stem, leaves, and grain. Ca^2+^ can increase the mechanical strength of cell walls, thereby fixing Cd in root cells and reducing Cd migration within the plant [39]. Ca^2+^ can neutralize the negative charge of the cell membrane, reducing the contact between harmful cations and the cell membrane [40]. 

In addition, the increased degree of Cd content in different parts of rice was the highest at Filling III. Cd content in rice grains had significantly negative effects on Mg^2+^ content in soil at Filling III (Table 6), indicating that Mg^2+^ played an important role in inhibiting Cd in rice at Filling III, respectively. Mg^2+^ is a necessary element for plant photosynthesis [20]. It was found that the photosynthesis of leaves was the highest at Filling III (Figure 4), which was the main source of carbohydrates in the grain and requires Mg^2+^ to participate in the photosynthesis process. There was an increased demand for Mg in rice, resulting in the absorption of a large amount of Mg^2+^ from the soil. Li found that Mg could promote plant growth in a Cd-contaminated environment, reduce the Cd concentration, and detoxify the physiological Cd toxicity in plants [8]. The Cd contents in the shoots and roots of Mg-deficient rice seedlings were higher than that of the normal-growth rice seedlings [41].

Karst areas are globally distributed and occupy about 15% of Earth’s surface [42]. There are abundant carbonate rocks in the southwestern region (e.g., Guangxi, Guizhou, and Yunnan Provinces) of China [43]. For example, Guizhou Province is the central area concentrated with exposed carbonate rocks in the southwest region, and the carbonate rock is widely distributed in an area of 130,000 km^2^, comprising 73% of the total area of the province [44]. There are differences between the karst area of LS and the non-karst areas of YS. There are the anomalies in the levels of high Cd in the soils of karst areas [45]. For example, the background of soil Cd is 0.659 mg·kg^−1^ in Guizhou, China [9]. The karst region was rich in CRIs, resulting in a lower bioaccumulation of Cd in rice. The natural phenomenon of carbonate rock dissolution in karst areas will be a strategy to treat the levels of high Cd in the soils and decrease Cd content of rice.

## 5. Conclusions

This study identified that the Ca^2+^, Mg^2+^, CO_3_^2−^/HCO_3_^−^, and OH^−^ of LS were significantly higher than that of YS, resulting in DTPA Cd of LS being lower than that of YS. Cd content in different parts of rice in YS was significantly higher than that of LS. The uptake of Cd from the soil during Filling III was critical in determining rice Cd accumulation. CRIs in the soil could affect Cd accumulation in rice. Ca^2+^ and Mg^2+^ had significant negative effects on Cd accumulation of rice at maturity and filling, respectively. CO_3_^2−^/HCO_3_^−^ and OH^−^ had significant negative effects on DTPA Cd in soil. In future studies, molecular biology, genomics, and other methods should be used further to investigate the effects and microbial mechanisms of carbonate rock dissolution (CRD) on Cd reduction in rice.

## Figures and Tables

**Table 1:** Basic physical and chemical properties of soils.

Soil Type	Cd	pH	Total Ca	Ca^2+^	Total Mg	Mg^2+^	CO_3_^2−^
	(mg·kg^−1^)		(g·kg^−1^)	(g·kg^−1^)	(g·kg^−1^)	(g·kg^−1^)	(mg·kg^−1^)
YS	0.67	5.85	3.25	0.55	1.72	0.43	10.76
LS	0.69	7.08	5.46	2.05	2.81	0.90	42.29

**Table 2:** DTPA Cd content of LS and YS in different treatments during rice filling and maturity (mg·kg^−1^).

Soil Type	Cd Level	Filling I	Filling II	Filling III	Maturity
LS	LS1	0.144 ± 0.032 ^g^	0.271 ± 0.017 ^f^	0.288 ± 0.015 ^f^	0.177 ± 0.025 ^f^
	LS2	0.547 ± 0.089 ^e^	0.585 ± 0.018 ^d^	0.654 ± 0.056 ^d^	0.542 ± 0.058 ^d^
	LS3	0.998 ± 0.013 ^c^	1.000 ± 0.030 ^c^	1.013 ± 0.072 ^c^	0.913 ± 0.082 ^c^
	LS4	1.754 ± 0.077 ^a^	1.986 ± 0.092 ^a^	2.053 ± 0.076 ^a^	1.864 ± 0.119 ^a^
YS	YS1	0.094 ± 0.012 ^g^	0.108 ± 0.021 ^g^	0.122 ± 0.018 ^g^	0.077 ± 0.022 ^f^
	YS2	0.405 ± 0.037 ^f^	0.426 ± 0.031 ^e^	0.473 ± 0.014 ^e^	0.350 ± 0.050 ^e^
	YS3	0.754 ± 0.020 ^d^	0.635 ± 0.025 ^d^	0.686 ± 0.036 ^d^	0.602 ± 0.052 ^d^
	YS4	1.462 ± 0.043 ^b^	1.372 ± 0.073 ^b^	1.432 ± 0.089 ^b^	1.351 ± 0.064 ^b^

The results of this study are expressed as mean (mg·kg^−1^) ± standard error (n = 3), superscripts (^a–g^) indicate significant differences between different categories of samples (LSD test, *p* < 0.05), and the same letters indicate no significant differences between samples.

**Table 3:** Total Ca and Mg content of LS and YS in different treatments during rice filling and maturity (g·kg^−1^).

Soil Type	Cd Level	Filling I		Filling II		Filling III		Maturity	
		Ca	Mg	Ca	Mg	Ca	Mg	Ca	Mg
LS	LS1	5.16 ± 0.12 ^a^	2.46 ± 0.13 ^a^	5.15 ± 0.21 ^a^	2.54 ± 0.20 ^a^	5.26 ± 0.07 ^a^	2.54 ± 0.29 ^a^	5.05 ± 0.18 ^a^	2.45 ± 0.15 ^a^
	LS2	4.90 ± 0.25 ^a^	2.24 ± 0.18 ^a^	5.03 ± 0.33 ^a^	2.46 ± 0.38 ^a^	5.02 ± 0.23 ^a^	2.33 ± 0.14 ^a^	5.13 ± 0.12 ^a^	2.33 ± 0.47 ^ab^
	LS3	4.73 ± 0.16 ^a^	2.13 ± 0.08 ^a^	4.92 ± 0.11 ^a^	2.24 ± 0.03 ^a^	4.93 ± 0.22 ^a^	2.20 ± 0.03 ^a^	4.58 ± 0.07 ^a^	2.13 ± 0.07 ^a^
	LS4	4.63 ± 0.01 ^a^	1.96 ± 0.04 ^a^	4.62 ± 0.23 ^a^	1.96 ± 0.07 ^a^	4.63 ± 0.16 ^a^	1.90 ± 0.04 ^a^	4.24 ± 0.16 ^a^	1.85 ± 0.02 ^a^
YS	YS1	2.86 ± 0.21 ^b^	1.53 ± 0.08 ^b^	2.84 ± 0.21 ^b^	1.57 ± 0.21 ^b^	3.03 ± 0.22 ^b^	1.74 ± 0.14 ^b^	2.92 ± 0.09 ^b^	1.73 ± 0.02 ^b^
	YS2	2.60 ± 0.13 ^bc^	1.45 ± 0.10 ^bc^	2.69 ± 0.04 ^bc^	1.45 ± 0.18 ^bc^	2.30 ± 0.18 ^bc^	1.48 ± 0.08 ^bc^	2.35 ± 0.09 ^bc^	1.46 ± 0.37 ^bc^
	YS3	2.50 ± 0.03 ^c^	1.25 ± 0.11 ^bc^	2.51 ± 0.02 ^c^	1.24 ± 0.06 ^bc^	2.22 ± 0.52 ^bc^	1.20 ± 0.03 ^bc^	2.70 ± 0.24 ^bc^	1.23 ± 0.03 ^bc^
	YS4	2.04 ± 0.08 ^c^	1.05 ± 0.04 ^c^	2.06 ± 0.16 ^c^	1.09 ± 0.04 ^c^	1.99 ± 0.14 ^c^	1.00 ± 0.05 ^c^	2.09 ± 0.10 ^c^	0.90 ± 0.20 ^c^

The results of this study are expressed as mean (mg·kg^−1^) ± standard error (n = 3), superscripts (^a–c^) indicate significant differences between different categories of samples (LSD test, *p* < 0.05), and the same letters indicate no significant differences between samples.

**Table 4:** Total Ca and Mg content of rice plants in different treatments at maturity (mg·kg^−1^).

Soil Type	Cd Level	Root		Stem		Leave		Grain	
		Ca	Mg	Ca	Mg	Ca	Mg	Ca	Mg
LS	LS1	1226 ± 141	2323 ± 75	2079 ± 160	2942 ± 126	1980 ± 21	1769 ± 67	294 ± 52	954 ± 153
	LS2	1402 ± 96	2446 ± 92	2253 ± 62	3083 ± 290	2042 ± 21	2087 ± 48	321 ± 61	1051 ± 44
	LS3	1520 ± 78	2876 ± 181	2386 ± 88	3462 ± 208	2159 ± 71	2057 ± 45	287 ± 54	1205 ± 154
	LS4	1895 ± 225	3075 ± 57	2800 ± 105	3613 ± 35	2201 ± 172	2347 ± 194	276 ± 48	1155 ± 96
YS	YS1	948 ± 118	1953 ± 153	2056 ± 72	2480 ± 152	1747 ± 37	1351 ± 113	394 ± 80	779 ± 140
	YS2	1134 ± 174	2238 ± 122	2290 ± 54	2701 ± 45	1799 ± 123	1644 ± 80	336 ± 50	1015 ± 101
	YS3	1379 ± 105	2401 ± 102	2788 ± 72	3058 ± 125	1898 ± 97	1961 ± 5	327 ± 69	1038 ± 37
	YS4	1659 ± 125	2625 ± 55	3021 ± 235	3427 ± 179	2051 ± 72	2163 ± 128	251 ± 5	1091 ± 48

The results of this study are expressed as mean (mg·kg^−1^) ± standard error (n = 3).

**Table 5:** Correlation between CRIs and DTPA Cd in soil.

	DTPA Cd	Ca^2+^	Mg^2+^	Total Alkalinity	pH
DTPA Cd	1	−0.347 *	−0.335 *	−0.320 *	−0.346 *
Ca^2+^	−0.147	1	0.212	0.746 **	0.817 **
Mg^2+^	−0.195	0.212	1	0.566 **	0.498 **
Total alkalinity	−0.220	0.746 **	0.566 **	1	0.966 **
pH	−0.246	0.817 **	0.498 **	0.966 **	1

** The correlation was significantly different at 0.01 level (two-tailed); * the correlation was different at 0.05 level (two-tailed).

**Table 6:** Correlation between Ca^2+^ and Mg^2+^ content in soil and Cd content in different parts of rice.

Pearson Correlation Coefficient		Root	Stem	Leaf	Grain
Ca^2+^	Filling stage I	−0.211	−0.332	−0.377	−0.240
	Filling stage II	0.010	−0.243	−0.320	0.000
	Filling stage III	−0.122	−0.301	−0.320	−0.356
	Maturity	−0.353	−0.596	−0.516	−0.779 *
Mg^2+^	Filling stage I	−0.438	−0.623	−0.631	−0.444
	Filling stage II	−0.307	−0.561	−0.620	−0.335
	Filling stage III	−0.599	−0.722 *	−0.777 *	−0.769 *
	Maturity	−0.095	−0.341	−0.271	−0.495

* The correlation was different at 0.05 level (two-tailed).

## Data Availability

Data will be made available on request.
